# Integrated Strategies for Controlling Water Cut in Mature Oil Fields in Kazakhstan

**DOI:** 10.3390/polym17070829

**Published:** 2025-03-21

**Authors:** Zhanat Alisheva, Kazim Nadirov, Ahmed N. Al-Dujaili, Gulmira Bimbetova, Zhanna Nadirova, Manap Zhantasov, Nurbol Tileuberdi, Ansagan Dauletuly

**Affiliations:** 1 Department of Geography and Environmental Sciences, Al-Farabi Kazakh National University, Al-Farabi Avenue 71, Almaty 050040, Kazakhstan; alishevazh9@gmail.com; 2 Department of Petroleum Engineering, M. Auezov South Kazakhstan University, Tauke Khan Avenue 5, Shymkent 160012, Kazakhstan; nadirovkazim@mail.ru (K.N.); gulmnaz@mail.ru (G.B.); zhanna.nadirova@inbox.ru (Z.N.); manapjan_80@mail.ru (M.Z.); 3 Petroleum Engineering Department, Amirkabir University of Technology, Tehran 11369, Iran; ahmed.noori203@aut.ac.ir; 4 Department of Hydrogeology, Engineering and Oil and Gas Geology, Satbayev University, Satbayev St. 22a, Almaty 050013, Kazakhstan; 5 Institute of Geological Sciences of K.I. Satpayev, Kabanbay Batyr Street 69, Almaty 050013, Kazakhstan; dauletuly.ansagan@mail.ru

**Keywords:** Kumkol and East Kumkol fields, polymer flooding, water cut, recovery factor, well-intervention, polymer retention, optimization

## Abstract

This study analyzed the physical and hydrodynamic characteristics of various horizons in the Kumkol and East Kumkol oil fields by special core analysis to integrate strategies for controlling water cuts and well-intervention procedures for a more effective oil flow rate in mature oil fields in Kazakhstan. The results indicated that the recovery factor (RF) for Horizon I is 48.3% (98.7% water cut), while Horizon II has an RF of 45.5% (97.9% water cut). Horizon III has an RF of 52.7% (98.8% water cut), and Horizon IV has an RF of 32.6% (98.6% water cut) in the Kumkol Field. In the East Kumkol Field, Horizon I has an RF of 49.5% (96.7% of water cut), and Horizon II has an RF of 31% (94.9% of water cut). The average increase in oil flow rate from well optimization ranges from 5.3 to 6.4 tons per day in the Kumkol Field and 5.22 tons per day in the East Kumkol Field. The maximum increase in oil flow rate is 10.8 tons/day for Horizon I in the Kumkol Field and 6.9 tons/day for Horizon II in the East Kumkol Field. The well-intervention procedures are more effective in the Kumkol Field than in the East Kumkol Field. Given the high water cut observed in these mature reservoirs, this study also examines polymer flooding as an enhanced oil recovery (EOR) technique to improve oil displacement efficiency and reduce water production. Polymer flooding has been successfully implemented in high water-cut reservoirs, including the Uzen field in Kazakhstan, demonstrating its ability to modify fluid filtration profiles and enhance oil recovery. The feasibility of applying polymer flooding in the Kumkol and East Kumkol fields is analyzed, along with a comparison of its effectiveness against conventional water shut-off and well-intervention methods. Additional research is needed to assess polymer retention, reservoir compatibility, and the economic feasibility of large-scale implementation.

## 1. Introduction

In the oil and gas extraction process, the production of unwanted water, known as water cut or water shut-off, remains one of the most difficult situations to manage and overcome [1]. In fact, effective control of water cut is of utmost importance since oil and gas are characterized by their non-renewable character, and the demand for energy is more and more considerable all over the world [2]. Furthermore, reducing environmental deterioration and enhancing hydrocarbon recovery rely on the efficiency of water management in oil fields [3,4,5]. Moreover, dealing efficiently with water cut is economical. Precisely, this can prevent huge unwanted expenses provoked by enormous unwanted water production in the oil industry [6]. In general, as noted in [7], oil wells must be abandoned unexpectedly, leading to a loss of productivity when undesirable water production is excessive. It is more than necessary to strengthen strategies that gradually lead to fairer control of water cut in mature oil fields. Typically, undesirable water is mainly caused by leaking tubing or casings from aging oil fields [8]. It is worth mentioning that large water cuts usually come from mature oil fields [9,10]. Since mature oil fields contribute significantly to global oil production [11], there is great interest in exploring and developing them.

A relevant literature review reveals that many strategies are usually developed or employed to manage water cut in aging oil fields. They are mostly mechanical and chemical and aim to gradually improve the efficiency of water evacuation. However, each of these strategies has its peculiarities and limitations. For instance, Ref. [9] utilized diversion wells as a technology to boost oil recovery in reservoirs facing enormous water shut-off [12]. However, this strategy is best suited for oil reservoirs located in fault blocks [13,14]. To diminish water intrusion into oil reserves and thus enhance recovery up to 5%, Ref. [15] believed that a low permeable barrier can be implemented by low salinity water in the short term. However, the applicability of such a method requires the existence of in situ movable fines, which are contained within the reservoir of interest. In order to better control the Chinese Changqing oil field, an interesting method consisting of characterizing waterflood direction and front has been proposed by [16]. According to them, such a method can increase the oil productivity of a given reservoir by 40% because it considerably reduces water cuts for producers. Nonetheless, such a method requires strong mobilization since the waterflood direction and front direction must be characterized using numerical and statistical techniques. As part of deep learning techniques, Long Short-Term Memory (LSTM) is proposed by [17] to forecast the productivity of oil fields at the stage of ultra-high water shut-off. Such a procedure relies on a huge amount of relevant data to provide good accuracy. In their aim to mitigate the exaggerated water content reduction in aging oil fields, Ref. [18] address reservoir heterogeneity by adequately applying bulk polymer gels. Although gel treatments are generally economical, they are most effective in fractured reservoirs where carbonate rocks are predominantly common. Flow control valves can be used as a strategy to control high water content in aging oilfields, as pointed out in [19]. However, this strategy does not provide optimized results since it leads to reduced oilfield production. Recently, to mitigate excessive water cut in mature oil fields, a novel nanomaterial reinforced particle gel (NRPG) has been developed by [20]. However, this strategy needs a special mechanism of polymerization with strong adaptability.

In addition to mechanical and hydrodynamic methods for managing water cut, there has been a growing interest in chemical methods for enhancing oil recovery in recent years, particularly polymer flooding. In Kazakhstan, previous attempts have been made to apply polymers in the Uzen field, and studies have been conducted to evaluate the effectiveness of polymer flooding in high water-cut reservoirs. The use of polymers allows for the modification of fluid filtration profiles in the reservoir, reducing water mobility and improving oil displacement efficiency. Given the high water cut observed in mature fields like Kumkol, further research is warranted to assess the feasibility and potential efficiency of polymer flooding as an advanced enhanced oil recovery (EOR) technique for optimizing reservoir performance and mitigating excessive water production.

In Kazakhstan, managing water cut is challenging, especially in mature fields like Tengiz Karachaganak [21], Uzen [7], and Akhmetzhan [22]. For example, the North Buzachi field has seen a drop in heavy oil production due to high viscosity and inefficiencies after 20 years of cold-water flooding [23]. Attempts with water shut-off, polymer flooding, and perforation adjustments have shown limited success. Gas-Steam Composite Stimulation (GSCS), successfully tested in Mortuk, increases sweep efficiency to 40% and oil recovery to 33.4%, compared to 12% with water flooding, and reduces water cut from 97% to 50%, making it a promising cost-effective solution [24,25].

This article outlines a comprehensive strategy to significantly reduce the high water cut observed in aging oil fields within the Kumkol oilfield in Kazakhstan. The proposed approach addresses the challenges of mature reservoirs by incorporating advanced technologies, optimized water management practices, and customized solutions to enhance oil recovery while minimizing water production issues.

### Theoretical Justification of Polymer Flooding

Polymer flooding is a widely recognized enhanced oil recovery (EOR) technique that involves injecting a polymer solution, such as polyacrylamide, into the reservoir to increase the viscosity of the displacing fluid and reduce its mobility ratio. This process effectively improves the sweep efficiency by mitigating water channeling through high-permeability zones and ensuring a more uniform displacement front [26].

One of the critical factors determining the success of polymer flooding is the ability of the injected polymer to maintain its viscosity under varying reservoir conditions, particularly temperature fluctuations. At elevated temperatures, polymer degradation and viscosity loss can significantly impact displacement efficiency, necessitating the selection of thermally stable polymers. The following table presents experimental data on the viscosity behavior of polymer solutions across different temperature ranges, providing insights into the thermal stability of potential polymer candidates for the Kumkol reservoir.

The viscosity of polymer solutions is highly dependent on temperature, as higher temperatures can lead to polymer degradation and reduced efficiency in improving the mobility ratio. To assess the thermal stability of the selected polymer system, viscosity measurements were conducted at different temperatures. The results, summarized in Table 1, illustrate the decline in polymer viscosity as the temperature increases, highlighting the need for thermally stable polymers in high-temperature reservoirs such as Kumkol.

As shown in Table 1, polymer viscosity decreases significantly with rising temperature, with a nearly 70% reduction observed between 30 °C and 80 °C. This trend underscores the necessity of selecting temperature-resistant polymer formulations to ensure the efficiency of polymer flooding under the reservoir conditions of the Kumkol Field.

It is important to note that the viscosity values presented in Table 1 were measured using a rotational rheometer (Anton Paar MCR series) at a constant shear rate of 10 s⁻^1^. This controlled shear rate ensures that the recorded viscosity reflects only the temperature-dependent behavior of the polymer solution, minimizing the influence of shear-thinning effects. At low polymer concentrations (≤1000 ppm), the solutions exhibit near-Newtonian behavior, making the viscosity-temperature relationship valid under these conditions. However, at higher concentrations, shear-thinning effects become more pronounced, requiring a more detailed rheological characterization for practical field applications.

For industry-standard applications, viscosity values are reported in centipoise (cP), with corresponding SI unit equivalents (Pa·s) provided in parentheses for reference. The conversion factor used is 1 cP = 0.001 Pa·s.

To optimize the polymer flooding process, laboratory experiments were conducted to determine the optimal polymer concentration for maximizing the oil displacement factor. The experimental results, summarized in Table 2, indicate that the highest oil recovery factor is achieved at a polymer concentration of 0.15–0.20% (previously expressed as 1500–2000 ppm), beyond which additional increases provide negligible economic or technical benefits.

Given the temperature sensitivity of polymer solutions (Table 1) and the observed recovery efficiency at different concentrations (Table 2), selecting a polymer formulation that balances viscosity retention at high temperatures and optimal concentration levels is crucial for effective polymer flooding.

To further refine polymer selection, additional laboratory tests were conducted to assess the performance of different polymer types under reservoir conditions. The key characteristics of the tested polymers are summarized below (Table 3):

Optimal polymer concentration: 0.10–0.15%
Reduction in water mobility under reservoir conditions: 35–50%
Increase in oil displacement efficiency: 15–20%
Optimal polymer solution viscosity for Kumkol conditions: 10–15 cP.

Among the tested polymers, nanoparticle-enhanced polymers exhibited the highest improvement in oil displacement efficiency, increasing the recovery factor by 25–30%. This suggests that nanoparticle-based polymers are a promising choice for the Kumkol reservoir, particularly given their enhanced stability and superior water mobility reduction properties. These findings highlight the potential of advanced polymer formulations to optimize polymer flooding strategies and maximize oil recovery under the given reservoir conditions.

## 2. Geological and Hydrocarbon Potential of the Kumkol and East Kumkol Deposits

### 2.1. Geological Setting

**Figure 1:**
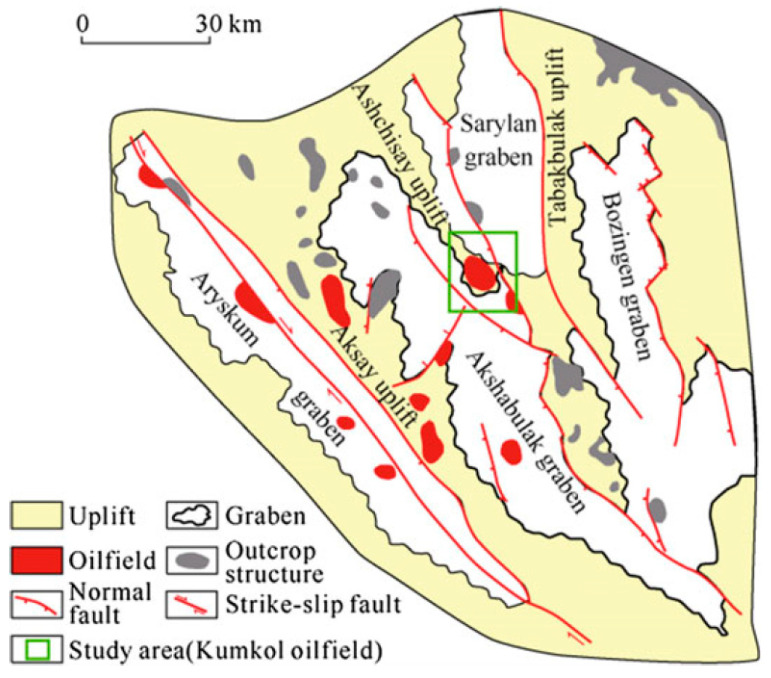
Structural location of Kumkol oilfield [30].

The Kumkol and East Kumkol oil fields are located within the Ashisay structural system of Paleozoic horst-anticlines, positioned in the Aryskum trough of the South Turgay depression [27] (Figure 1). This region forms a part of the Turan tectonic plate and is characterized by its complex geological formations, which include a series of uplifted blocks and folded anticlines [28]. These structural features play a critical role in hydrocarbon accumulation and have made the area a significant contributor to regional oil production [29].

To assess the feasibility of polymer flooding, Table 4 presents the key geological and physical parameters of the Kumkol reservoir, including formation water salinity, oil viscosity, and other critical reservoir characteristics.

These reservoir conditions are highly favorable for polymer flooding implementation, as the process can significantly reduce water saturation in productive zones and enhance the oil displacement efficiency.

**Figure 2:**
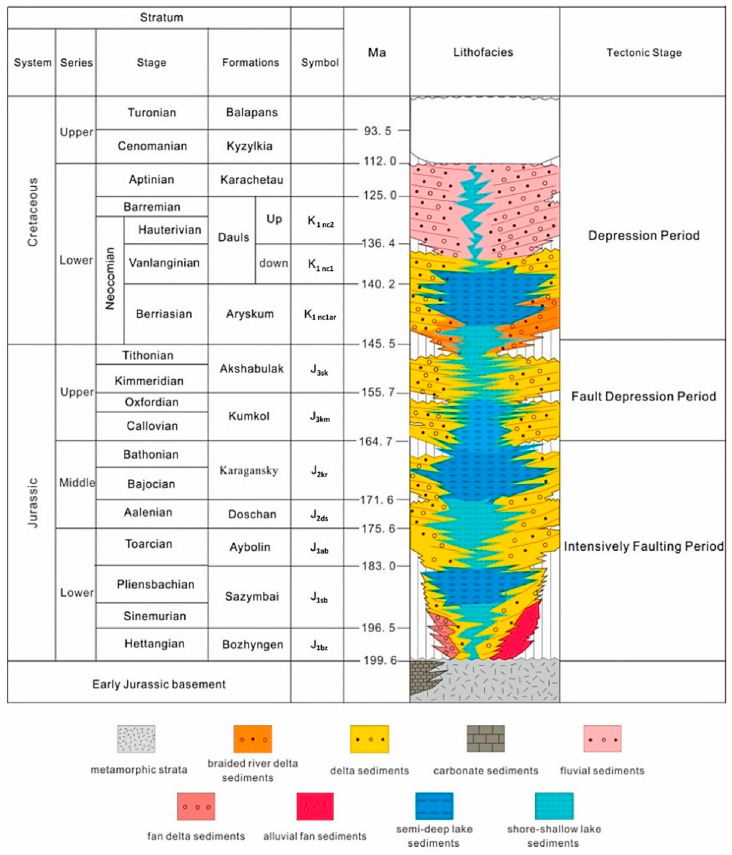
Stratigraphic column map of the South Turgay Basin [35].

The South Turgay Basin features an Early Mesozoic basement and upper unconformity sedimentary layers. These comprise a Jurassic rift system and a Cretaceous quaternary post-rift depression sedimentary system [31] (Figure 2). During the Early–Middle Jurassic, lacustrine mudstone dominated the sedimentary layers, while the lateral margins of the graben often transitioned into coarse-grained gravel and estuarine clastic rocks [32,33,34]. The estuarine deposits, which are widely distributed, originated from the uplifted basement along the lateral margins of the graben [35]. At the Jurassic–Cretaceous boundary, rift-related sedimentation occurred as tectonic activity in the grabens subsided [36]. The basin shifted into a phase of slow subsidence, with localized denudation in some areas. This process gave rise to the basal sandstone of the Cretaceous, known as the Aryskum Formation [37]. Deltaic sediments were extensively deposited during the basin’s filling phase, while fluvial systems were more prominent in the northern parts of the South Turgay Basin [38]. During this time, river systems expanded, lake levels declined, and the water bodies gradually migrated southward. Semi-deep lake deposits are also evident in the Bozingen Graben [39].

The geological framework comprises metamorphosed Paleozoic-Proterozoic basement rocks overlain by Mesozoic and Cenozoic sedimentary layers [40]. The basement features sericite-quartzite schists, quartzite, and hydromica-kaolinite clays, with thicknesses ranging from 16 to 267 m. Overlying Mesozoic formations include Jurassic and Cretaceous terrigenous deposits. The Jurassic system features productive horizons J-I through J-IV within the Doschanskaya and Kumkol suites [41], while the Cretaceous system includes the Aryskum horizon in the Daul formation, which contains two main oil-bearing zones (M-I and M-II) [42] (Figure 2).

**Figure 3:**
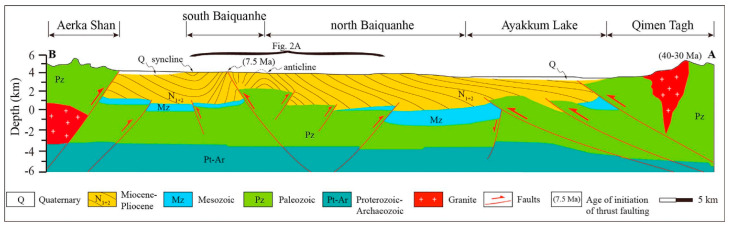
Hydrocarbon potential units of the Kumkol basin [13].

The Kumkol deposit exhibits commercial hydrocarbon potential across multiple horizons:

1. Horizon M-I: A layered, arched oil deposit with dimensions of 14.2 × 4.6 km and a height of 48.7 m.
2. Horizon M-II: A massive oil deposit, 5.2 × 2.7 km in size and 25 m high.
3. Horizon J-I: Contains the largest oil-bearing area with a layered, arched deposit measuring 19.7 × 8.8 km and 133.3 m in height (gas cap: 43.8 m; oil zone: 89.5 m).
4. Horizon J-II: Features a layered, arched deposit with dimensions of 18 × 7.8 km and oil-bearing and gas-bearing areas of 61,249 and 2728 thousand m^2^, respectively.
5. Horizon J-III: Comprises three deposits, including a gas-capped oil deposit and two smaller accumulations, with oil-bearing areas up to 38,083 thousand m^2^.
6. Horizon J-IV: A lithologically limited gas and oil deposit, 4.0 × 5.8 km in size and 37.3 m in oil thickness.
7. Horizon PZ_1_-PR: Includes four deposits, with oil and gas caps, hosted in quartz-sericite schists and quartzites, exhibiting oil-saturated thicknesses of 0.8 to 12.5 m (Figure 3 and Table 5).

The heterogeneity of horizons and reservoir beds is characterized by sandiness and dissection coefficients [43]. Table 5 provides data on reservoir bed thickness, average values, and variations, along with reservoir properties and oil saturation. These metrics are derived from GIS and core analysis.

### 2.2. Hydrocarbon Potential

The field’s recoverable reserves are estimated at 54,278 thousand tons, classified under categories A, B, and C1. These classifications are based on rigorous geological and geophysical analysis, with category A representing the highest degree of certainty [44]. This substantial reserve base underscores the Kumkol Field’s importance in meeting regional energy demands and sustaining long-term production targets.

As of recent evaluations, the cumulative production from the Kumkol Field has reached approximately 43,200 thousand tons, which equates to 79.6% of the initial recoverable reserves [45]. This impressive recovery rate reflects the implementation of advanced extraction technologies and efficient reservoir management practices [46]. However, as production nears the economic limit of recoverability, challenges arise in accessing the remaining reserves. Strategies such as enhanced oil recovery (EOR) techniques are being considered to optimize extraction [47].

This study was achieved due to a late stage of development for the field, which has declining oil production. The main reasons are a significant reserve depletion degree, a high water cut of the extracted products, and complete field drilling. Since the beginning of development in 2017, oil production at the asset has amounted to 552.2 thousand tons. Cumulative oil production at both fields was 43,200 thousand tons, or 79.6% of the initial recoverable reserves, with a water cut of 98.4%.

## 3. Data and Methods

The physical and hydrodynamic characteristics were determined based on the results of special core analysis for 1173 samples from different horizons of Kumkol and East Kumkol oil fields (Table 6 and Table 7). The average petrophysical properties (permeability, porosity, and initial oil saturation) were determined [48,49] based on the results of core analysis from different horizons of the Kumkol Field (Table 7) and the East Kumkol oil field (Table 8).

The characteristics of surface tension, wetting angle, and fluid densities identified using core analysis are listed in Table 9.

**Figure 4:**
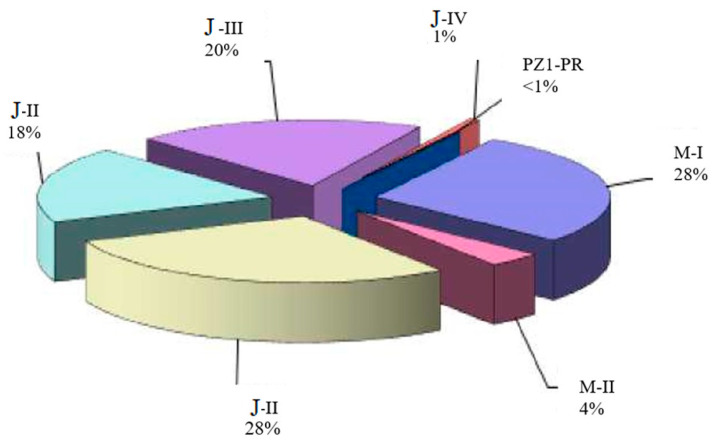
Distribution of initial geological oil reserves by layers of the Kumkol Field.

The hydrocarbon reserves of the Kumkol Field were first calculated in 1987. The last calculation of oil reserves was performed in 2012, and gas and condensate reserves in 2008. In the same year, the reserves of the PZ1-PR formation were promptly reviewed (within the territory of activity of Turgai). Almost all of the oil reserves at the Kumkol Field are classified as industrial, with less than 1% of the total reserves classified as non-industrial. Most oil reserves are concentrated in the M-I and J-I formations (28–29% each) (Figure 4).

**Figure 5:**
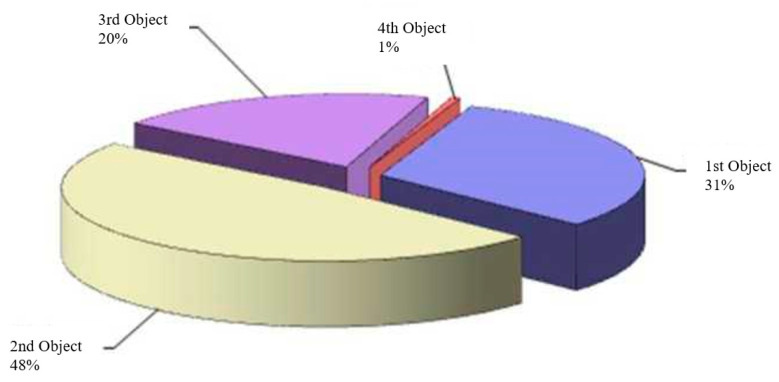
Distribution of initial recoverable oil reserves by development sites of the Kumkol Field.

The II development object is the main one regarding the volume of recoverable oil reserves (48%) (Figure 5).

**Figure 6:**
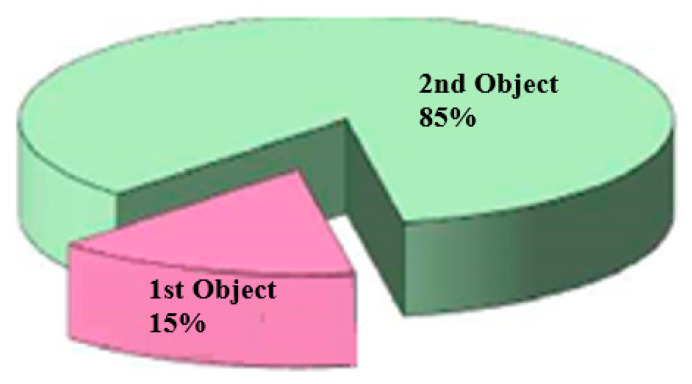
Distribution of initial recoverable oil reserves by development sites of the East Kumkol Field.

The hydrocarbon reserves of the Vostochny Kumkol deposit were last approved in 2013. The initial geological/recoverable oil reserves of the East Kumkol Field in the contract territory of JSC Turgai-Petroleum amount to 4786/2022 thousand tons, and the initial geological/recoverable reserves of dissolved gas amount to 80/32 million m^3^, and the presence of gas caps has not been established. The main one regarding the volume of recoverable oil reserves (85%) is the II development object (Figure 6), which includes the J-I and J-II layers. Thus, most of the reserves are concentrated in the Jurassic deposits.

**Figure 7:**
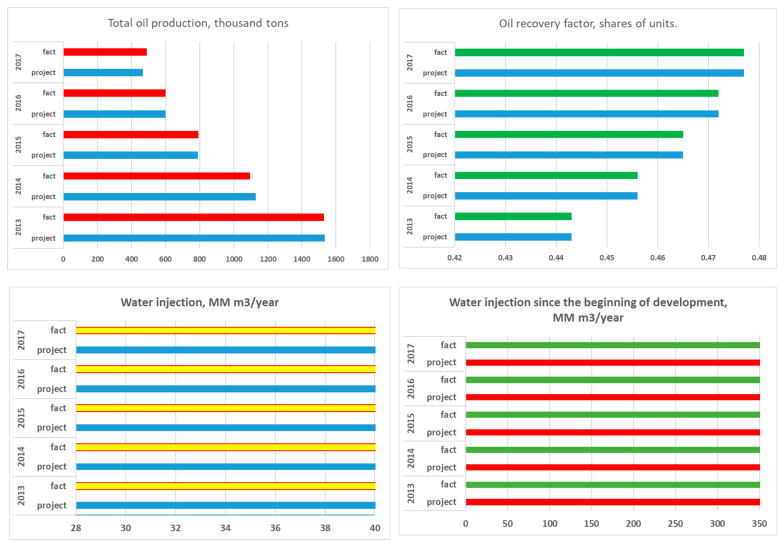
Production and injction data of the Kumkol deposit for the period between 2013 and 2017.

**Figure 8:**
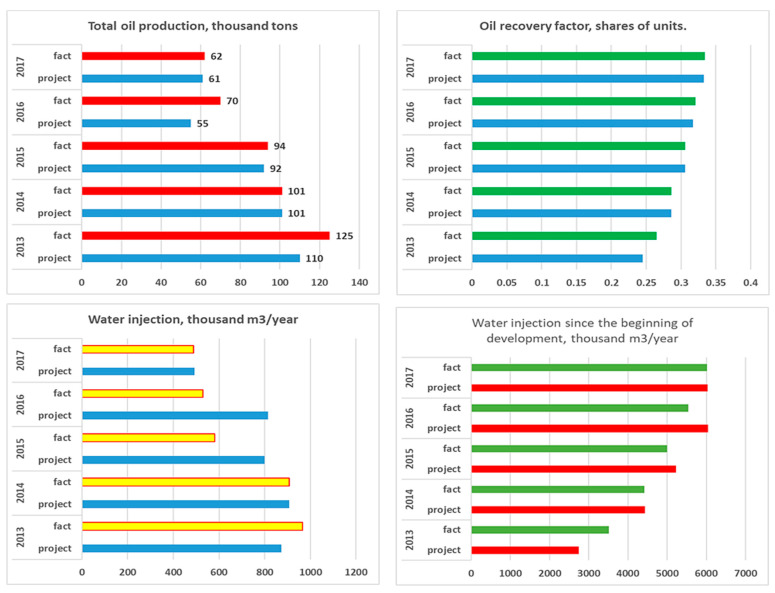
Production and injection data of the East Kumkol deposit for the period between 2013 to 2017.

Production and injection data of the Kumkol deposit and East Kumkol deposit for the period under study between 2013 to 2017 are illustrated in Figure 7 and Figure 8, respectively.

The residual recoverable hydrocarbon reserves of the field as of 01.01.2018 are (geological/recoverable): oil—45.550/10.653 thousand tons in category A + B + C1, 58/35 thousand tons in category C2 gas dissolved in oil—4428/1 170 million m^3^ in category A + B + C1 and 0.4/0.2 million m^3^ in the category (Table 10 and Table 11).

The residual oil reserves (geological/recoverable) at the field amount to 3189/425 thousand tons, and dissolved gas in oil amounts to 58.1/10 million m^3^. All reserves are classified as ABC1 (Table 12 and Table 13).

## 4. Results and Discussion

According to the core data description, the reservoirs at the field are represented by sandstones, sandy rocks with insignificant layers of clay rocks and clays, and less often siltstones, with a low content of carbonate cement. Sandstones of Neocomian deposits are usually fine-grained and often very poorly cemented. In the deposits of the Kumkol suite, the sandstones are also fine-grained and poorly cemented, especially in the Yu-I and Yu-II horizons. There are fine-medium-grained, in thin interlayers—coarse-medium-grained sand varieties. In the deposits of the Doshchanskaya suite, the amount of medium-grained and coarse-medium-grained varieties increases. The lithological and petrophysical characteristics of the Pz + PR deposits with the addition of core sampling from well 5014 have not changed. The rocks are represented by pyroxene andesite porphyrites, with significant secondary changes of the greenstone metamorphism type, quartzite, weathered to varying degrees, and schists. The field is at a late stage of development with declining oil production. The main reasons are a significant degree of reserves depletion, characterized by high water cut of the extracted products, and complete field drilling. Since the beginning of development, 41,603 thousand tons of oil have been produced at the field, or 79.6% of the NIR. All production units, except object IV, are developed with the reservoir pressure maintenance by reinjecting the produced water into the reservoir. Since the beginning of development, 327,970 thousand m^3^ of water have been injected into the reservoirs. Current compensation is 101%, accumulated—91.7%.

### 4.1. Horizon Object I—Kumkol Field

**Figure 9:**
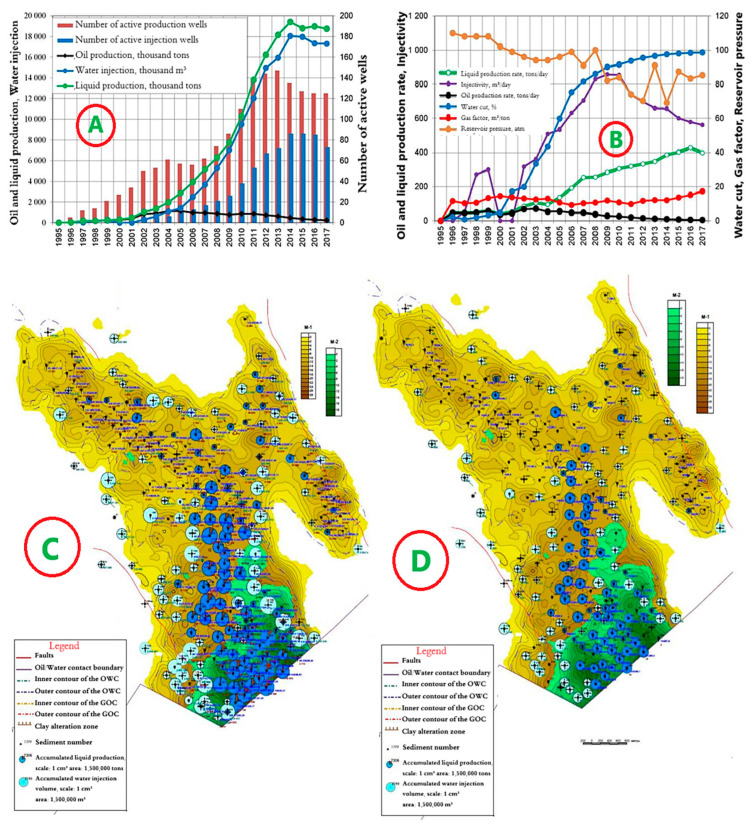
(**A**,**B**) Dynamics of the main development indicators. (**C**) Map of accumulated selections. (**D**) Map of current selections for Object I in the Kumkol Field.

The development system is marginal flooding. The distance between wells is 250 m. The dynamics of the main development indicators are presented in Figure 9A,B.

Since the beginning of development, 13,369 thousand tons of oil, or 82.0% of the reserve, have been produced. A total of 145,648 thousand m^3^ of water has been pumped into the formations. The current oil flow rate is 4.9 tons/day, and the liquid flow rate is 397.3 tons/day. Well injectivity is 560.9 m^3^/day. Current compensation is 96%, accumulated—89%. The current recovery factor is 0.483, with a water cut of 98.7%. Maps of the current state of development and accumulated withdrawals for object I are shown in Figure 9C,D.

### 4.2. Horizon Object II—Kumkol Field

**Figure 10:**
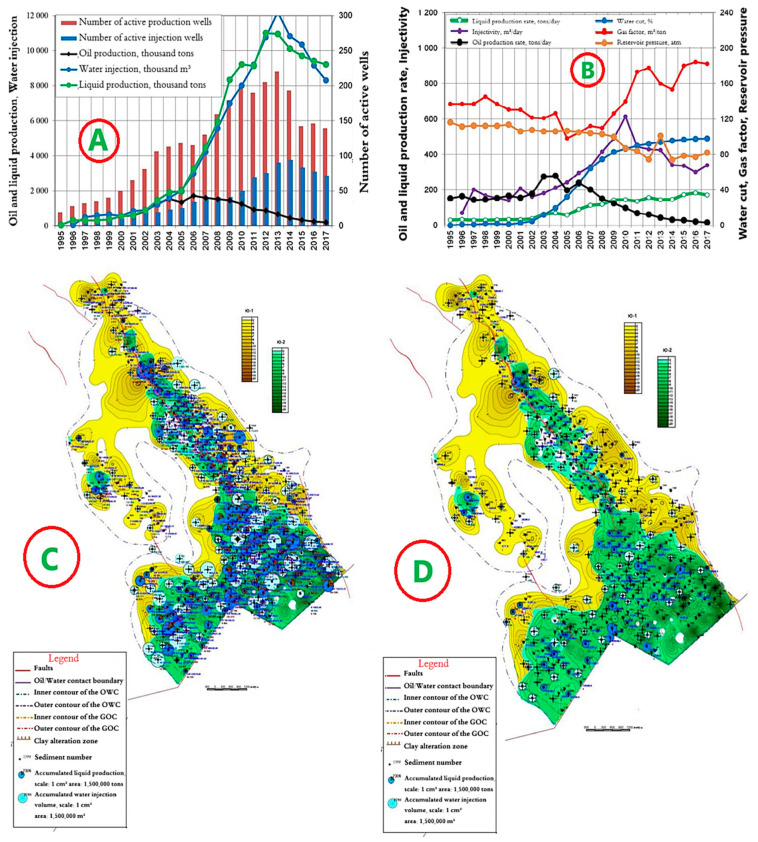
(**A**,**B**) Dynamics of the main development indicators. (**C**) Map of accumulated selections. (**D**) Map of current selections for Object II in the Kumkol Field.

The development system is a 9-point area impact system, with a distance between wells of 250 m. The dynamics of the main development indicators are presented in Figure 10A,B.

Since the beginning of development, 18,802 thousand tons of oil have been produced at the site, or 74.3% of the reserve. A total of 108,412 thousand m^3^ of water has been injected into the formations. The current oil flow rate is 3.4 tons/day, liquid—170.8 tons/day. Well injectivity is 338.8 m^3^/day. Current compensation is 93%, accumulated—92%. The current oil recovery factor is 0.455, with water cut of 97.9%. Maps of the current state of development and accumulated selections for object II are shown in Figure 10C,D.

### 4.3. Horizon Object III—Kumkol Field

**Figure 11:**
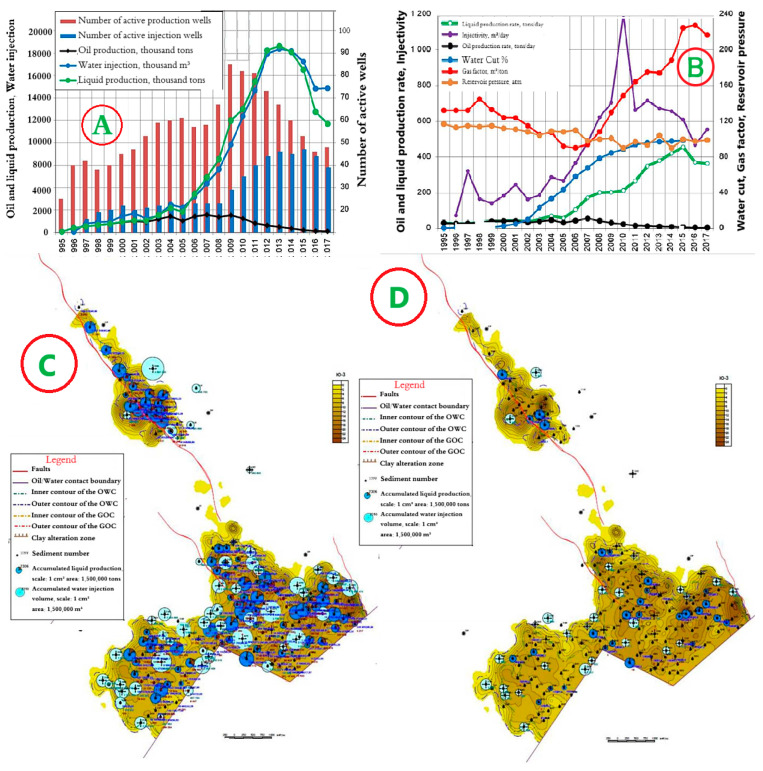
(**A**,**B**) Dynamics of the main development indicators. (**C**) Map of accumulated selections. (**D**) Map of current selections for Object III in the Kumkol Field.

The development system is a 9-point area impact system, with a distance between wells of 250 m. The dynamics of the main development indicators are presented in Figure 11A,B.

Since the beginning of development, 9209 thousand tons of oil, or 88.9% of the reserve, has been produced at the site. A total of 73.579 thousand m^3^ of water has been injected into the formations. The current oil flow rate is 4.1 tons/day, liquid—363.3 tons/day. Well injectivity is 554.1 m^3^/day. Current compensation is 138%, accumulated—98%. The current oil recovery factor is 0.527, with water cut of 98.8%. Maps of the current state of development and accumulated withdrawals for site III are shown in Figure 11C,D.

### 4.4. Horizon Object IV—Kumkol Field

**Figure 12:**
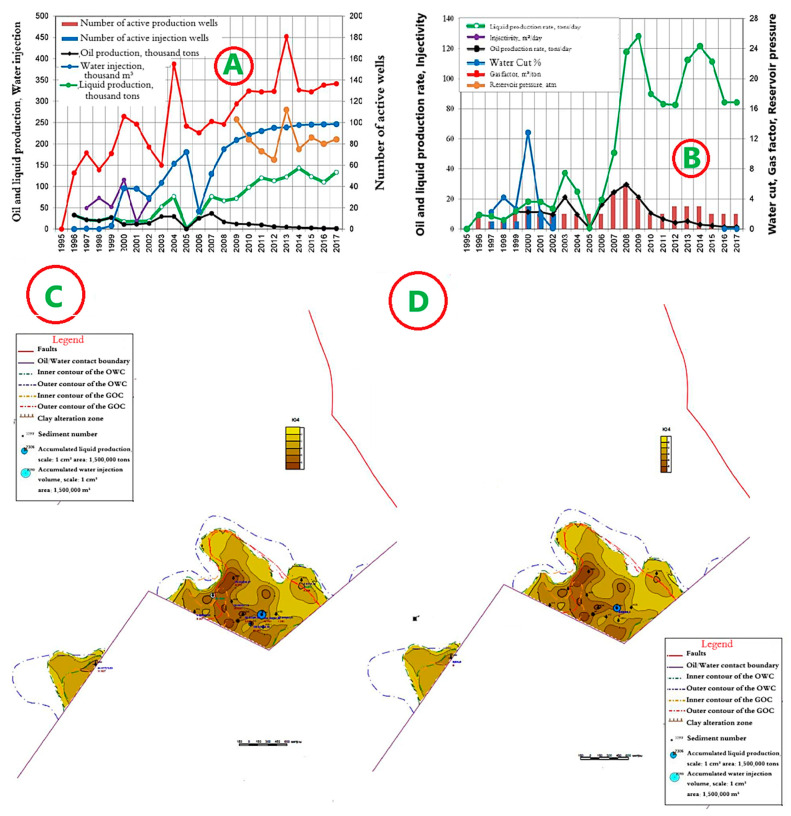
(**A**,**B**) Dynamics of the main development indicators. (**C**) Map of accumulated selections. (**D**) Map of current selections for Object IV in the Kumkol Field.

The development system is a natural regime with selective placement of wells. The distance between wells is 500 m. The dynamics of the main development indicators are presented in Figure 12A,B.

Since the beginning of development, 223 thousand tons of oil, or 77.4% of the reserve, has been produced at the facility. The current oil flow rate is 1.4 tons/day, liquid—133.3 tons/day. Current compensation is 0%, accumulated—24%. The current recovery factor is 0.326, with a water cut of 98.6%. Maps of the current state of development and accumulated withdrawals for facility IV are shown in Figure 12C,D.

The observed difference in retention factors between Horizon III and Horizon IV is primarily due to variations in reservoir properties. Horizon III exhibits higher permeability (634 mD) and porosity (23%), allowing for better fluid retention and displacement efficiency. In contrast, Horizon IV has significantly lower permeability (145 mD) and porosity (21.5%), leading to reduced oil retention and increased water encroachment. Additionally, structural differences contribute to this variation, as Horizon IV is located in a lower structural zone, making it more prone to water influx from underlying aquifers.

### 4.5. Horizon Object I—East Kumkol Field

**Figure 13:**
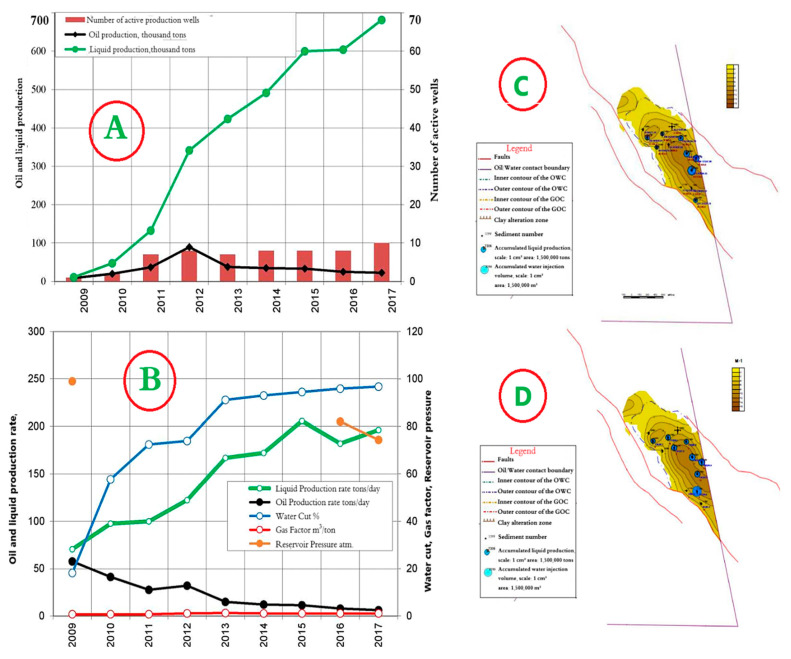
(**A**,**B**) Dynamics of the main development indicators, (**C**) Map of accumulated selections. (**D**) Map of current selections for Object I in the East Kumkol Field.

The development system is a natural depletion regime with a well spacing (400 × 400 m). The dynamics of the main development indicators are presented in Figure 13A,B. Since the beginning of development, 308 thousand tons of oil have been produced at the facility, or 101% of the reserve. The accumulated liquid production amounted to 3331 thousand tons. The current recovery factor is 0.495, with a water cut of 96.7%. The current oil flow rate is 6.1 tons/day, liquid—196.2 tons/day.

As can be seen from Figure 13A,B, oil production decreased in 2013, which is associated with an increase in water cut from 74% to 91%. Maps of the current state of development and accumulated production for object I are shown in Figure 13C,D.

### 4.6. Horizon Object II—East Kumkol Field

**Figure 14:**
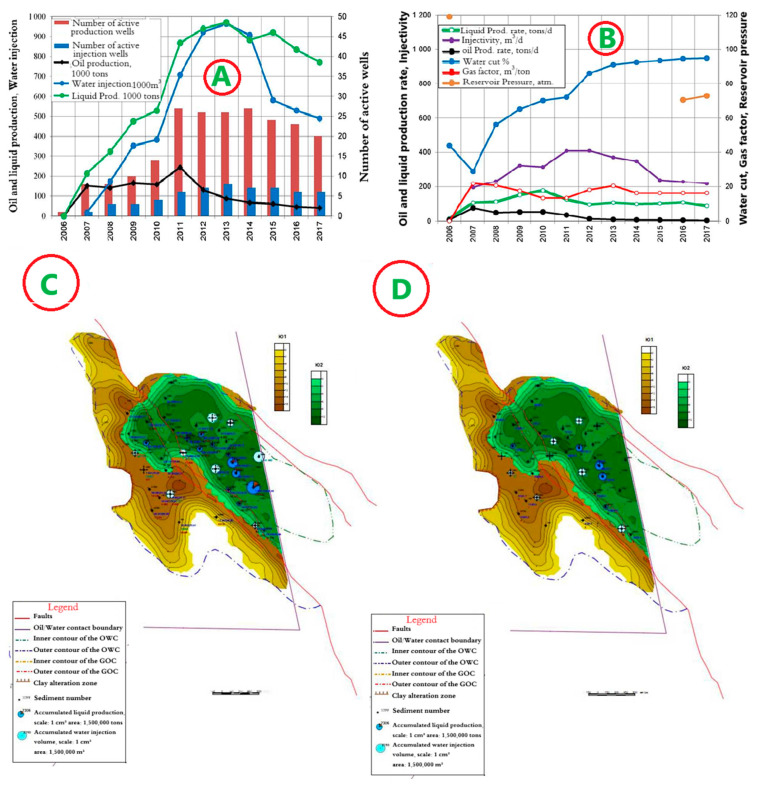
(**A**,**B**) Dynamics of the main development indicators. (**C**) Map of accumulated selections. (**D**) Map of current selections for Object II in the East Kumkol Field.

The development system is a marginal flooding system with a distance between wells of 400 m. The dynamics of the main development indicators are presented in Figure 14A,B. Since the start of development, 1289 thousand tons of oil, or 75.1% of the reserve, has been produced at the site. A total of 6023 thousand m^3^ of water has been injected into the formations. Current oil flow rates are 4.0 tons/day, liquid—86.3 tons/day, and well injectivity is 217.2 m^3^/day. Current compensation is 65.2%, accumulated—76.7%. The current oil recovery factor is 0.310, with water cut of 94.9%. Maps of the current development status and accumulated production are shown in Figure 14C,D.

Oil production at the asset amounted to 552.2 thousand tons in 2017. Cumulative oil production at both fields is 43,200.0 thousand tons, or 79.6% of the initial recoverable reserves, with a water cut of 98.4%. The availability of industrial category reserves within the licenses at the 2017 production level is 20 years. Over the past 5 years, the number of operating production wells has decreased by 123 units due to the transfer of wells to the inactive fund for injection and control and observation wells to monitor the energy state of deposits. The reasons for the inactivity of production wells are low unprofitable flow rates and high water cuts of the product. The fund for operating injection wells has decreased by 9 units due to the transfer of wells to the inactive fund.

### 4.7. Well Interventions for Kumkol Field

**Figure 15:**
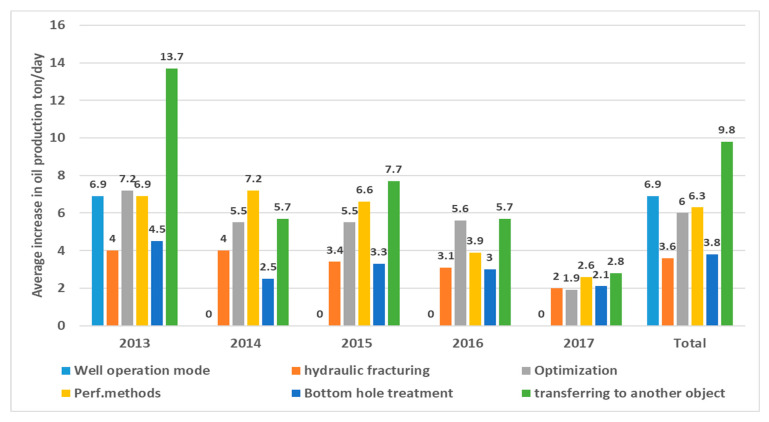
Well interventions for Kumkol Field between 2013 and 2017.

The proposed and conducted main types for the well interventions in this study include optimization, optimizing well operating modes, various perforation methods (such as additional perforations, re-perforations, and inclusions), transferring wells to different sites, the bottom hole zone treatment, and performing hydraulic fracturing. Over the 5 years of the simulation, 132 events have been completed to optimize the well operating mode, of which 127 wells were operated with a positive effect. At the same time, a gradual decrease in the efficiency of this type is noted—the average increase in oil flow rate decreased over this period from 7.2 tons/day (2013) to 1.9 tons/day (2017). On average, for 2013–2017, the increase in oil was 6 tons/day (Figure 15 for Kumkol Field).

**Figure 16:**
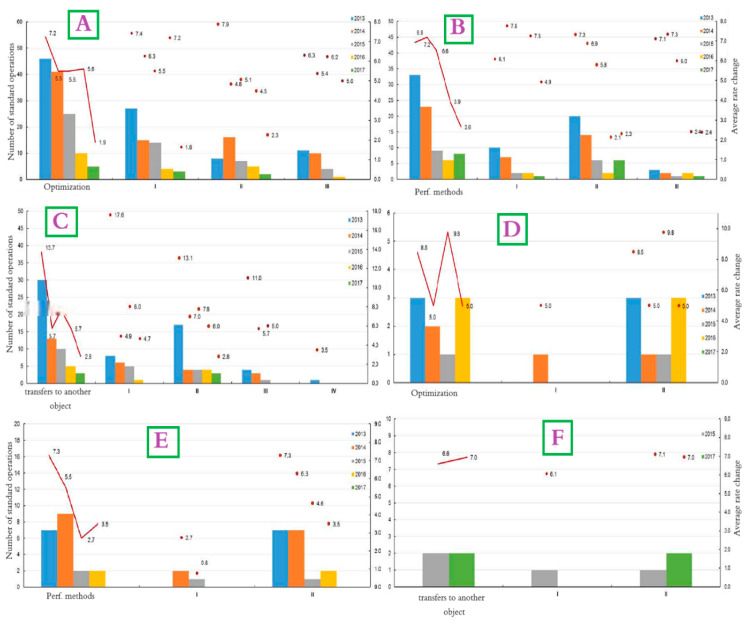
The successful dynamics of objects between 2013 and 2017, 1. The Kumkol Field for (**A**) well optimization operations, (**B**) perforation methods, (**C**) transfer to another object II. The East Kumkol Field for (**D**) well optimization operations, (**E**) perforation methods, (**F**) transfer to another object.

Concerning operational facilities, the well optimization is consistent across the board, with the average increase in oil flow rate ranging from 5.3 to 6.4 tons per day, depending on the horizon (Figure 15). Figure 16A illustrates the successful dynamics of well optimization operations in the Kumkol Field. However, there is a noticeable decline across all facilities related to the perforation method, as depicted in Figure 16B for the Kumkol Field. The maximum increase in oil flow rate according to well transfers to another object over the 5 years is observed at development horizon I and amounts to 10.8 tons/day. The increase in oil flow rate at development horizon II amounted to 9.8 tons/day, at horizon III—8.4 tons/day, and at Horizon IV—3.5 tons/day (Figure 15 and Figure 16C). Figure 16C shows the dynamics of successful operations on transfers to another horizon. The effectiveness of the event is decreasing.

It should be noted that, based on the magnitude of the increase in oil flow rate, this type of well intervention is the most effective in the conditions of the Kumkol Field.

### 4.8. Well Interventions for East Kumkol Field

**Figure 17:**
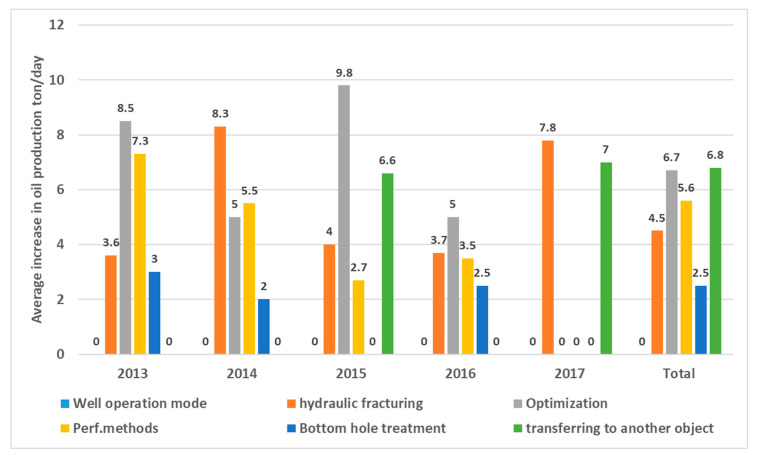
Well interventions for East Kumkol Field between 2013 and 2017.

Over the past 5 years, the primary types of well interventions implemented in the field include the optimization of well operating modes, various perforation methods (such as additional perforations, re-perforations, and inclusions), transferring wells to different sites, the bottom hole zone treatment, and performing hydraulic fracturing. Forty-seven successful operations were analyzed to assess the effectiveness of measures for increasing oil production between 2013 and 2017. The primary types of well interventions during this period included perforation methods (43%), treatment of the bottom hole zone (23%), and optimization of well operating modes (19%) (Figure 17).

During the analyzed period, the operating mode of nine wells in the field was optimized, resulting in positive outcomes for all of them. The average increase in oil flow rate decreased to five tons per day in 2016. Over the period from 2013 to 2017, the average increase was 6.7 tons per day. Concerning specific operational objects, the highest increase in oil flow rate due to well optimization occurred at horizon II, with an increase of 6.9 tons per day. Meanwhile, the increase in flow rate at the development horizon was five tons per day. Figure 16D illustrates the trends in successful well optimization operations.

The highest increase in oil flow rate observed from the perforation methods over the simulated years was for horizon II, reaching 6.3 tons per day. In contrast, horizon I had an increase of 2.1 tons per day. Figure 16E illustrates the dynamics of successful operations using perforation methods, showing a noticeable decline in the operation’s effectiveness over time. Four transfers to another horizon were carried out during the analyzed period, resulting in an increase of 6.8 tons per day in the average oil flow rate. Figure 16F shows the dynamics of successful operations for these transfers.

## 5. Economic and Technological Feasibility of Polymer Flooding

To objectively assess the economic viability of various enhanced oil recovery (EOR) methods, a comparative cost-benefit analysis was conducted. This evaluation considered both capital expenditures (CAPEX) and operational expenditures (OPEX) associated with different water cut management strategies, including traditional mechanical methods and polymer flooding. The results, summarized in Table 14, provide a direct comparison of the cost per cubic meter of oil produced and the corresponding increase in oil recovery for each method.

The data indicate that polymer flooding offers the most cost-effective solution, delivering a higher oil recovery rate at a lower cost per cubic meter compared to both hydraulic fracturing (HF) and water shut-off treatments.

Unlike hydraulic fracturing, which involves high initial costs and is often less effective in reservoirs with advanced water breakthrough, polymer flooding provides a more controlled displacement process, improving sweep efficiency and delaying water breakthrough. Similarly, while water shut-off treatments can temporarily mitigate excessive water production, they do not provide long-term improvements in oil displacement efficiency.

By enhancing mobility control and increasing the volumetric sweep efficiency, polymer flooding achieves a more sustainable oil production rate while minimizing operational costs. This makes it a highly attractive EOR strategy for mature fields like Kumkol, where the high water cut (98.4%) necessitates an efficient method to improve oil displacement while maintaining economic feasibility.

In addition to technical advantages, the return on investment (ROI) for polymer flooding is significantly higher than that of alternative methods, given its lower cost per unit of oil recovered and long-term operational benefits. The ability to reduce excessive water production and extend the productive life of the reservoir further supports the economic feasibility of implementing polymer flooding in the Kumkol Field.

## 6. Conclusions

The study highlights the critical challenges posed by high water cut in the mature reservoirs of the Kumkol and East Kumkol fields, where water saturation exceeds 98% in several productive horizons. Traditional water shut-off methods and well interventions have demonstrated limited efficiency in mitigating excessive water production, necessitating the implementation of advanced chemical-enhanced oil recovery (EOR) techniques, particularly polymer flooding.

Hydrodynamic modeling and experimental results confirm that polymer flooding is the most viable method for enhancing oil recovery in high-water-cut reservoirs. Laboratory studies indicate that the optimal polymer concentration lies within the 0.15–0.20% range (previously expressed as 1500–2000 ppm), providing a 15–20% increase in the oil displacement factor while effectively controlling water mobility. Furthermore, numerical simulations demonstrate that temperature stability is a key factor, as polymer viscosity declines significantly at temperatures above 60 °C, necessitating the selection of thermally stable polymer formulations.

The comparative cost analysis further supports the economic feasibility of polymer flooding. Unlike hydraulic fracturing and water shut-off treatments, polymer flooding exhibits a superior balance between cost and oil recovery efficiency, delivering higher incremental recovery at a lower cost per cubic meter of produced oil. Given the high water cut and the declining efficiency of traditional well interventions, polymer flooding presents a technically and economically justified solution for maximizing oil recovery in mature reservoirs.

Strategic Implications and Recommendations

1. Implementation of polymer flooding as the primary EOR strategy in high-water-cut reservoirs to improve mobility control and enhance sweep efficiency.
2. Optimization of polymer selection based on formation temperature, water salinity, and polymer rheology to ensure long-term stability and efficiency.
3. Integration of polymer flooding with existing water management strategies to minimize water production and extend the economic life of the reservoir.
4. Continued monitoring and numerical modeling to evaluate the long-term performance of polymer flooding and adjust injection strategies accordingly.

In conclusion, polymer flooding represents the most effective and sustainable method for mitigating excessive water production and enhancing oil recovery in the Kumkol and East Kumkol fields. The findings of this study provide a strong technical and economic foundation for the large-scale deployment of polymer-based EOR solutions in Kazakhstan’s mature oil fields.

## Figures and Tables

**Table 1:** Viscosity reduction of polymer solution at different temperatures.

Temperature (°C)	Polymer Solution Viscosity (cP)	Polymer Solution Viscosity (Pa·s)
30	25	0.025
40	20	0.020
50	15	0.015
60	12	0.012
70	10	0.010
80	8	0.008

**Table 2:** Determination of the optimal polymer concentration for Kumkol Field.

Polymer Concentration (ppm/%)	Oil Recovery Factor (%)
500	40
1000 (0.10%)	50
1500 (0.15%)	55
2000 (0.20%)	57
2500 (0.25%)	58

**Table 3:** Laboratory analysis of polymer characteristics and their effect on oil recovery.

Polymer Type	Solution Viscosity (cP)	Increase in Oil Recovery Factor (%)
Polyacrylamide	10–15	15–20
Crosslinked Polyacrylamide	20–30	20–25
Polymer with Nanoparticles	30–40	25–30

**Table 4:** Reservoir characteristics.

Parameter	Value
Average Reservoir Temperature	52–60 °C
Formation Water Salinity	49.784 mg/L
Oil Viscosity	1–10 cP
Reservoir Permeability	100–500 mD
Porosity	18–22%
Water Cut	98.4%
Oil Recovery Factor (ORF)	35–50%

**Table 5:** Statistical indicators of heterogeneity of productive horizons of the Kumkol deposit.

Horizon, Object	Number of Wells Used for Determination	Sandiness Coefficient, Units		Coefficient of Dismemberment, Fractions of Units		Distribution Coefficient, Fractions of Units
		Average	Variations	Average	Variations	
M-I	628	0.621	0.048	2.70	0.123	1
M-II	440	0.756	0.022	7.70	0.213	1
J-I	931	0.540	0.175	4.07	0.214	0.997
J-II	765	0.712	0.086	2.11	0.216	0.976
J-III	610	0.777	0.032	2.24	0.229	0.954
J-IV	188	0.465	0.316	2.55	0.380	0.977
PZ-PR	100	0.491	0.365	3.14	0.235	0.929

**Table 6:** Number of core sampling for each horizon in the East Kumkol oil field.

Parameter/Horizon	M-I	M-II	J-I	J-II	J-III	J-IV	Total	P.Z.
Number of samples	95	132	378	257	241	52	1155	95

**Table 7:** Characteristics of reservoir properties and oil saturation of Kumkol Field.

Horizon	Method	Name	K, 10^−3^ µm^2^	φ, Fraction	Soi, Units
M-I	Laboratory Research cores	Average value	2584	0.293	
		Coefficient of variation	0.82263	0.00026	
	Geophysical Research wells	Average value		0.26	0.56
		Coefficient of variation		0.006	0.041
M-II	Laboratory Research cores	Average value	1337	0.266	
		Coefficient of variation	1.3043	0.0158	
	Geophysical Research wells	Average value		0.27	0.57
		Coefficient of variation		0.001	0.034
J-I	Laboratory Research cores	Average value	313	0.242	
		Coefficient of variation	3.9669	0.0338	
	Geophysical Research wells	Average value		0.23	0.61
		Coefficient of variation		0.077	0.048
J-II	Laboratory Research cores	Average value	1891	0.264	
		Coefficient of variation	7.9627	0.03141	
	Geophysical Research wells	Average value		0.25	0.64
		Coefficient of variation		0.008	0.05
J-III	Laboratory Research cores	Average value	634	0.234	
		Coefficient of variation	2.5279	0.0216	
	Geophysical research wells	Average value		0.25	0.64
		Coefficient of variation		0.016	0.05
J-IV	Laboratory Research cores	Average value	145.9	0.215	
		Coefficient of variation	5.5997	0.0323	
	Geophysical Research wells	Average value		0.21	0.54
		Coefficient of variation		0.134	0.029
PZ + PR	Laboratory Research cores	Average value	0.2	0.077	
		Coefficient of variation			
	Geophysical Research wells	Average value		0.14	0.54
		Coefficient of variation		0.02	0.029

**Table 8:** Characteristics of reservoir properties and oil saturation of East Kumkol Field.

Horizon	Determination Method	Name	Permeability, 10^−3^ µm^2^	Porosity, Fractions of Units	Initial Oil Saturation, Units
M-I	Laboratory Research cores	Average value	1.8	0.289	
		Coefficient of variation	0.039–6.323	0.204–0.336	
	Geophysical Research wells	Average value		0.26	0.51
		Coefficient of variation		0.013	0.015
J-I	Laboratory Research cores	Average value	0.23	0.239	
		Coefficient of variation	0.024–1.082	0.192–0.281	
	Geophysical Research wells	Average value		0.21	0.58
		Coefficient of variation		0.035	0.034
J-II	Laboratory Research cores	Average value	3.952	0.277	
		Coefficient of variation	0.015–12.041	0.179–0.327	
	Geophysical Research wells	Average value		0.24	0.59
		Coefficient of variation		0.022	0.031

**Table 9:** Characteristics of surface tension, wetting angle, and fluid densities.

Parameter	Terms and Conditions	Surface Tension (a), Dyne/cm	Wetting Angle (θ), °	Density of Oil, kg/m^3^	Density of Water, kg/m^3^
Water–gas	Laboratory	72	0		
Oil–water	Laboratory	30	30	660	1051
Oil–water	Layer	30	30	660	1051

**Table 10:** Status of oil reserves at the Kumkol Field as of 1 January 2018.

Objects	Initial Oil Reserves, Thousand Tons				Current Reserves, Thousand Tons			
	On the State Balance Sheet							
	Geological		Extractable		Geological		Extractable	
	ABC1	C2	ABC1	C2	ABC1	C2	ABC1	C2
I/MI + II	27.685	-	16.302	-	14.316	-	2933	-
II/J-T + J-II	41.300	58	25.308	35	22.497	58	6506	35
III/J-III	17.484	-	10.358	-	8275	-	1149	-
IV/J-IV+ (PZ_1_- PR)	684	-	288	-	461	-	65	-
Total	87.153	58	52.256	35	45.550	58	10.653	35

**Table 11:** Status of dissolved gas reserves of the Kumkol Field as of 1 January 2018.

Objects	Initial Reserves of Dissolved Gas, Million m^3^				Current Reserves, Million m^3^			
	On the State Balance Sheet							
	Geological		Extractable		Geological		Extractable	
	ABC1	C2	ABC1	C2	ABC1	C2	ABC1	C2
I/MI + II	340	-	200	-	184	-	44	-
II/J-T + J-II	5454	0.4	3341	0.2	3048	0.4	935	0.2
III/J-III	2290	-	1357	-	1100	-	167	-
IV/J-IV+ (PZ_1_- PR)	126	-	54	-	96	-	24	-
Total	8210	0.4	4953	0.2	4428	0.4	1170	0.2

**Table 12:** Status of oil reserves at the East Kumkol Field as of 1 January 2018.

Objects	Initial Oil Reserves, Thousand Tons				Current Reserves, Thousand Tons			
	On the State Balance Sheet							
	Geological		Extractable		Geological		Extractable	
	ABC1	C2	ABC1	C2	ABC1	C2	ABC1	C2
I/MI	622	-	305	-	314	-	−2	-
II/J-T + J-II	4164	-	1717	-	2875	-	427	-
Total	4786	-	2022	-	3189	-	425	-

**Table 13:** Status of dissolved gas reserves of the East Kumkol Field as of 1 January 2018.

Objects	Initial Reserves of Dissolved Gas, Million m^3^				Current Reserves, Million m^3^			
	On the State Balance Sheet							
	Geological		Extractable		Geological		Extractable	
	ABC1	C2	ABC1	C2	ABC1	C2	ABC1	C2
I/MI	0.5	-	0.2	-	0.2	-	−0.1	-
P/UT + P	79.8	-	32	-	58	-	10	-
Total	80	-	32	-	58	-	10	-

**Table 14:** Comparative cost analysis of traditional methods vs. polymer flooding.

Method	Cost ($/m^3^ of Oil Produced)	Average Increase in Oil Recovery (%)
Hydraulic Fracturing (HF)	20–30	5–10
Water Shut-off Treatments	15–25	8–12
Polymer Flooding	10–15	15–20

## Data Availability

The original contributions presented in this study are included in the article. Further inquiries can be directed to the corresponding author.
